# Beyond neurotransmission: dopamine as an emerging biotic and abiotic stress regulator in plants

**DOI:** 10.1007/s44154-026-00291-8

**Published:** 2026-03-24

**Authors:** Devyani Shinde, Sunil Pareek

**Affiliations:** Department of Agriculture and Environmental Sciences, National Institute of Food Technology Entrepreneurship and Management, Plot No. 97, Sector 56, HSIIDC Industrial Area, Kundli, Sonepat, Haryana 131028 India

**Keywords:** Abiotic stress, Redox homeostasis, Chilling injury, Molecular mechanisms, Postharvest

## Abstract

Dopamine (DA) is a catecholamine that plays a role in both animals and plants. Popularly known as a neurotransmitter hormone in animals involved in motor control and reward sensing, it also has a defense-related function in the plant kingdom. In plants, DA functions as a redox-active metabolite, a signalling regulator, and a metabolic modulator, rather than a classical neurotransmitter. Accumulating evidence demonstrates that DA enhances tolerance to diverse abiotic and biotic stresses, including drought, salinity, heavy metals, nutrient imbalance, temperature extremes, and pathogen attack, by stabilizing photosynthetic machinery, optimizing root architecture, improving water and nutrient use efficiency, and activating antioxidant and detoxification systems. Mechanistically, DA operates through coordinated regulation of reactive oxygen species (ROS) signalling, calcium-mediated secondary messenger cascades, transcription factor activation (WRKY, ERF, NAC), and reprogramming of nutrient and ion transporter networks. DA also exhibits extensive crosstalk with phytohormones, fine-tuning growth defence trade-offs in a species and stress-specific manner. Collectively, these findings position DA as a central signalling hub integrating redox balance, metabolic plasticity, and transcriptional control in plants. Understanding the conserved structure yet divergent biosynthesis and signalling logic of DA across kingdoms provides critical insights into its distinct modes of action and highlights its potential as a next-generation regulator for enhancing plant resilience under changing environmental conditions.

## Introduction

Plants being sessile in nature, are required to continuously adapt to the diverse and often uncertain environment around them. Escalating climate change with each passing day, exerts a range of biotic stresses, like pathogens, pest attacks, and abiotic stresses like drought, salinity, and heavy metals on plants, challenging their normal life span (Mareri et al. 2022; Teshome et al. 2020; He et al. 2018). However, plants exhibit resilience to these conditions through their physiological, biochemical, and molecular adaptations tailored to the specific stress. These endogenous defence mechanisms often become insufficient under prolonged or severe stress conditions (Anjum et al. 2011). Thus, an external intervention is needed to reinforce plants defence and stress resilience.

In this context, signalling molecule plays a pivotal role by transducing the external stimuli into coordinated internal cellular responses. The recent emergence of signalling molecules has reshaped perspectives on stress mitigation strategies in plants (Choi et al. 2017). Different phytohormones and neurotransmitters, such as 1-methylcyclopropene (Djanaguiraman et al. 2011; Hussain et al. 2019), methyl jasmonate (Yu et al. 2018), epibrassinolide (Tanveer et al. 2019), and melatonin (Ayyaz et al. 2022) signals the adaptive mechanisms for mitigating plant stress. One such novel molecule from the family of catecholamines is dopamine (DA), which has gained importance for its multifunctional approach in alleviating stress (Chakraborty et al. 2022; Gao et al. 2020a; Liu et al. 2020a, 2025).

DA (3,4-dihydroxyphenethylamine) is a nitrogen-containing biogenic amine present in almost all plants; for instance, it has a high share in the pulp of yellow and red bananas (Bruno et al. 2013), plantains, and avocados (Kulma and Szopa 2007). Another catecholamine, such as norepinephrine, is distinctively found in ‘Peyote’ and ‘Dona Ana’ cacti, with lower amounts in apples, eggplant, oranges, tomatoes, peas, beans, and spinach (Kulma and Szopa 2007). The presence of DA in plants was first detected in ‘Saguaro’ cactus *(Carnegia gigantea),* where a spike in endogenous DA was observed within five minutes in the wounded tissue (Steelink et al. 1967; Kimaru et al. 1968). Further, the reasearchers have also proposed that environmental stimuli can instigate the production of DA in plant tissue as a protective response (Steelink et al. 1967). More recent studies have also documented the protective role of DA in drought (Gao et al. 2020a), salinity (Abdelkader et al. 2012), nitrate stress (Liu et al. 2020b), and also from biotic stress (Zhang et al. 2023c). DA in plants regulates carbohydrate metabolism, osmotic balance, antioxidant activity (Bala 2020), proline metabolism, enhances photosynthesis, modifies root architecture and net vegetative growth (Liu et al. 2025), as well as fosters resistance against bacterial and fungal threats. In association with plant stress research, DA has also been examined for mitigating postharvest losses of horticultural produce, paving the way for a broad-spectrum application of this biogenic amine (Aghdam et al. 2025; Ali et al. 2023; Nazari et al. 2024).

The present review aims to comprehensively synthesize current understanding of DA as an emerging multifunctional molecule in plant stress biology, encompassing its roles at physiological, biochemical, and molecular levels under diverse abiotic and biotic stresses. While DA is well-established as a neurotransmitter in animals, its regulatory role in plants remains relatively unexplored and scattered across isolated studies. This article seeks to bridge the gap by consolidating evidence on DA’s biosynthesis, signalling pathways, interaction with hormones, modulation of antioxidant defence, transporter regulation, and its novel application in mitigating postharvest chilling injury (CI). The novelty of this work lies in presenting DA not merely as antioxidant but as a central signalling hub influencing transcription factors, calcium signalling, ion homeostasis, and secondary metabolism. The review proposes DA as a potential next-generation stress regulator in both pre- and postharvest systems, and sets the stage for future molecular and translational research in plant stress physiology.

## DA: structure and biosynthesis in animals and plants

Despite sharing a similar molecular structure across the animal and plant kingdoms, DA performs completely different functions in both. Conventionally known to be a neurotransmitter hormone in animals, the emerging evidence indicates that DA can also act as a signalling molecule in plant stress mitigation, demonstrating impressive evolutionary flexibility. Therefore, elucidating the molecular structure and biosynthesis of DA is fundamental for understanding its distinct mode of action.

### Chemical structure and properties

Dopamine, epinephrine (adrenaline), and norepinephrine (noradrenaline) are the nitrogen-containing biogenic molecules of the catecholamine family. All three of them are derived from the same precursor called tyrosine, which is an amino acid (Kulma and Szopa 2007). The structure of DA is essentially a benzene ring featuring 2 adjacent hydroxyl (-OH) groups known as the catechol group, which is characteristic to catecholamines. In DA, this catechol group is attached to an ethylamine side chain, giving DA its name as a catechol-substituted phenethylamine. This molecular arrangement is structurally known as 3,4-dihydroxyphenethylamine (Nagatsu and Stjärnet 1997), IUPAC name 4-(2-aminoethyl) benzene-1,2-diol (Liu et al. 2020a) with molecular formula C₈H₁₁NO₂ and molar mass 153.18 g/mol. Here, the two -OH group and one primary amine (-NH_2_) are the functional groups, and phenethylamine serves as the backbone of DA (Liu et al. 2020a). Both functional groups can engage with water by hydrogen bonding, making DA a highly polar molecule, which is also reflected by its negative octanol–water partition coefficient (Rajnák et al. 2024). The presence of the -NH_2_ makes DA an organic base, allowing it to form salts in acidic conditions via protonation of the nitrogen atom’s lone pair of electrons (Dong et al. 2023). The antioxidant property of DA is a function of its catechol moiety (3,4-dihydroxyphenyl), which serves as a suitable reducing agent (Smolyaninov et al. 2018). The two -OH groups in the aromatic ring are the key to this reducing power (Burda and Oleszek 2001). These functional groups promote the electron/hydrogen donation and, in some cases, metal chelation to neutralize the free radical, exhibiting antiradical activities (Dimić et al. 2017).

### Role in animals: neurotransmission and motor control

The significance of DA was first recognized in animal systems, where it functions as a neurotransmitter. Although synthesized in 1910 by Barger and Ewins while studying the chemical structure and properties of biologically active amines (Hornykiewicz 1986, 2002). It was nearly five decades later, in 1957, that it gained recognition as a neurotransmitter rather than just an insignificant intermediate in noradrenaline biosynthesis (Marsden 2006). For this breakthrough, Arvid Carlsson was awarded the Nobel Prize in physiology and medicine in 2002. He demonstrated that intravenous administration of the precursor of DA, that is, 3,4-dihydroxyphenylamine (DOPA) reversed the akinetic effect of reserpine. This finding suggests that the depletion of DA is a key cause of the akinesia observed with reserpine in animals, confirming the crucial role of DA in motor control (Yeragani et al. 2010) contributing to the reflex action. DA also plays a significant role in animals by regulating social behaviour, reward processing, memory formation and consolidation, cerebral cognition, and attention (Nieoullon 2002).

As a chemical messenger, DA is stored in dopaminergic neurons in animals. Within the animal cell, DA operates in two different ways: one is fast and direct, called wiring, where the stored DA from neurons travels through the synaptic cleft via synaptic transmission (Costa and Schoenbaum 2022; Ozcete et al. 2024). Wiring operates on a millisecond scale, providing fast, direct, and localized signalling. For instance, in the nigrostriatal pathway, where DA travels from the substantia nigra to modulate striatal neurons and control movement (Ozcete et al. 2024). Another mode of action of DA other than wiring is volumetric transmission. It is an alternative method for DA signalling that shows a distinct, indirect, and slower diffusion-based allocation (Liu et al. 2021). Here, the extra synaptic diffusion occurs through D1-D5 receptors. This alternate route is important for the mesolimbic system, which contributes to reward processing and emotional regulation (Kawahata et al. 2024).

### Role in plants: stress response and redox buffering

The role of DA in plants takes a remarkable turn from that of animals. In plants, DA acts as a signalling molecule, helping them to survive against stress. The major roles performed by DA here include redox buffering, osmoregulation (Ahammed and Li 2023), allelochemical, metal chelation, and hormonal crosstalk (Cao et al. 2023b; Akcay et al. 2024; Akula and Mukherjee 2020; Guidotti et al. 2013). DA is a potent water-soluble antioxidant that exhibits the highest inhibitory scavenging activity against free radicals like diphenyl picrylhydrazyl (DPPH) (Kanazawa and Sakakibara 2000). It plays a role in the plant defence system by scavenging ROS like superoxide ion (O_2_^−^) and hydrogen peroxide (H_2_O_2_) and modulates the gene expression of antioxidant enzymes (Ali et al. 2023; Liu et al. 2020a). When compared to natural and artificial antioxidants, DA outperforms glutathione, ascorbic acid, catechin gallate, butylated hydroxy toluene, and butylated hydroxy anisole (Ahemmad and Li 2023).

The earliest study on the presence of catecholamines and other neurotransmitters, like serotonin, in bananas was carried out in year 1958 (Waalkes et al. 1958). This investigation led to a further inquiry where the presence of DA was confirmed in some common fruits and vegetables, such as banana peel and pulp, plantains, and avocados (Udenfriend et al. 1959). Furthermore, the sudden accumulation of DA in the wounded tissue of the saguaro cactus indicated its protective role in plants. However, it was not clear how this accumulation guarantees protection (Steelink et al.^1967^). Following this, the first fluorescence histochemical technique for determining catecholamine in plants was performed by Kimura in 1968. Since then, the research on DA’s role in plant stress and environmental adaptation has acquired momentum. Investigations have been done on DA’s role in various stress adaptations in plants, like N_2_ stress (Farouk et al. 2023), drought stress (Bandurska 2022; Gao et al. 2020b; Liang et al. 2018), heavy metal stress (Ahammed et al. 2025; Cao et al. 2023b; Prestes et al. 2025), salt stress, waterlogged condition (Cao et al. 2023a, 2024), and infection by *Peronosporaceae* spp. in cucumber (Ji et al. 2022) *Valsa mali* infection in apple (Liu et al. 2022a). DA also interacts with other hormones, such as progesterone (Akcay et al. 2024) and melatonin (Cao et al. 2024), exhibiting synergistic effects on plants' stress adaptation capabilities.

### Biosynthetic pathway

Biosynthesis of DA is a complex, dynamic, and enzyme-specific process that is sensitive to external and internal stimuli (Ahammed and Li 2023). It starts with a precursor tyrosine, in both animals and plants. However, the sources of tyrosine are different between these two. Animals obtain tyrosine either from phenylalanine or from dietary sources (Fernstrom and Fernstrom 2007). Whereas in plants, tyrosine is synthesized through the shikimate acid pathway (Marchiosi et al. 2020).

**Fig. 1:**
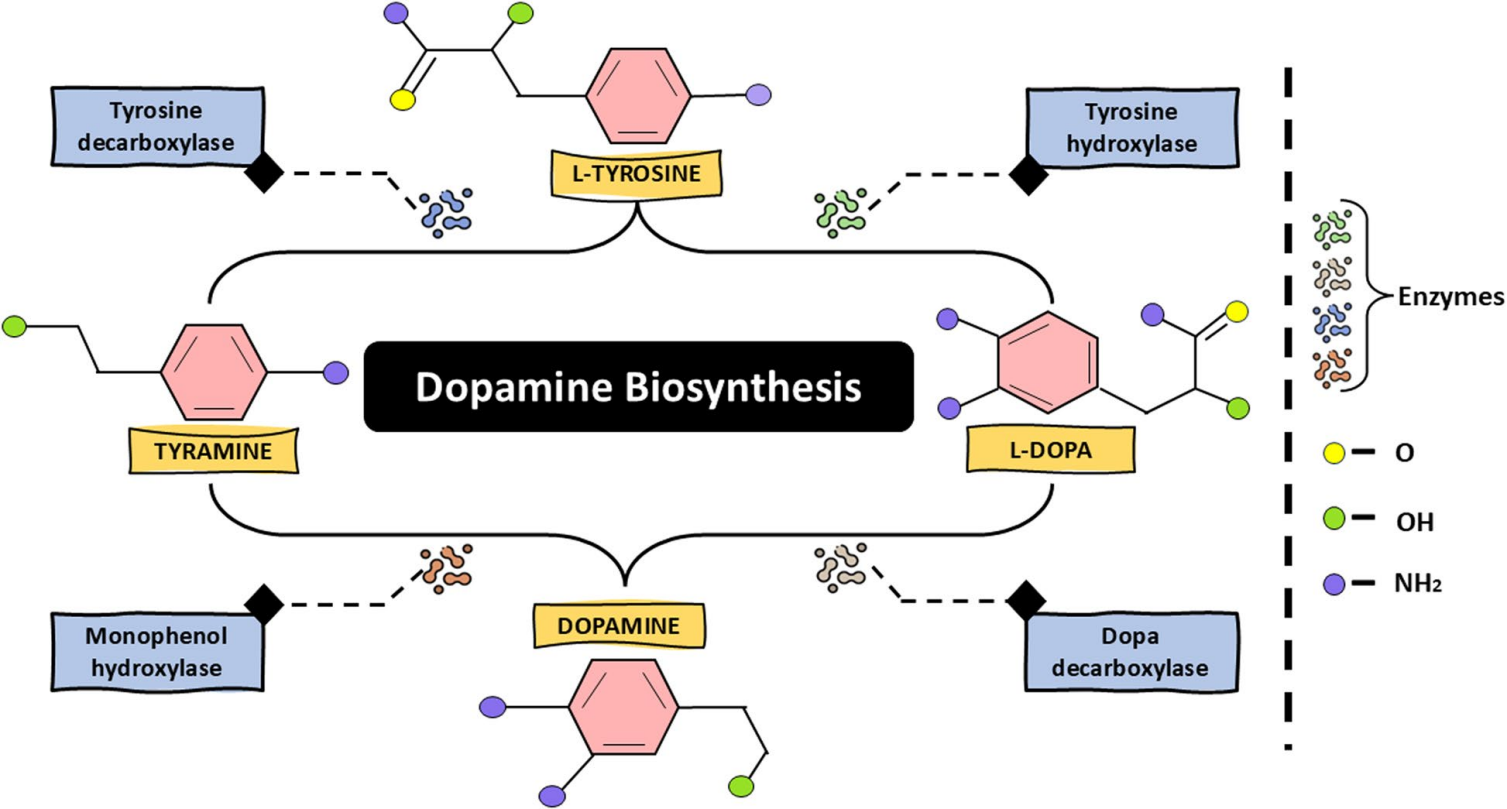
Dopamine biosynthesis in Plants: plants produce dopamine from L-tyrosine via two different enzymatic pathways. In one pathway, tyrosine decarboxylase removes a carboxyl group from L-tyrosine to form tyramine, which is then hydroxylated by monophenol hydroxylase to create dopamine. In the other pathway, tyrosine hydroxylase hydroxylates L-tyrosine to form L-3,4-dihydroxyphenylalanine, which is subsequently converted into dopamine by DOPA decarboxylase. Dashed arrows show enzyme-driven reactions, and colored symbols depict functional groups (–O, –OH, –NH₂) in the molecular structures

The biosynthesis of DA in animals takes place via a single route where the initial enzyme tyrosine hydroxylase acts as a rate-limiting enzyme (Daubner et al. 2011; Nagy and Hiripi 2002), whereas in plants as shown in Fig. 1, there are two alternate established pathways for DA synthesis (Ahammed and Li 2023). The first pathway in plants begins with the decarboxylation of tyrosine by the enzyme tyrosine decarboxylase (*TyDC*), which produces tyramine as an intermediate. This tyramine is then hydroxylated by monophenol hydroxylase (*MH*) to produce DA (Li et al. 2015). In the second alternative pathway, the intermediate form is levodopa (L-DOPA) rather than tyramine. This L-DOPA is obtained from the hydroxylation of tyrosine, facilitated by tyrosine hydroxylase (*TH*). This L-DOPA undergoes decarboxylation by DOPA decarboxylase (*DDC*), forming DA (Marchiosi et al. 2020). Thus, plants can produce DA via two alternative pathways; however, which pathway the plant opts for depends on the dominance and availability of enzymes, and sometimes it depends on the plant species (Ahammed and Li 2023; Kulma and Szopa 2007).

The biosynthesis of DA depends on external and internal stimuli, plant type, and injured or stressed tissue. The site of synthesis also differs as per the need; for instance, the tissue-level accumulation of DA takes place in roots (Ahmadi et al. 2014), leaves (Liang et al. 2018). In early growth, DA is synthesized in the young leaves at the rosette stage in opium poppy (*Papaver somniferum*) and declines as the plant begins flowering (Kamo and Mahlberg 1984). Along with the different sites of localization at the tissue level, the amount of DA production also varies. Many studies have shown a diverse range of production, ranging from a few nanograms to a considerable amount of micrograms, depending on the species (Kulma and Szopa 2007).

The subcellular localization of DA in plants is not as extensively characterized as in animals. Furthermore, the studies focusing on where DA builds up in the plant cell point towards the vacuole, which acts as a storage compartment, whereas, small quantity of DA has also been found in the cytoplasm; however, whether the cytoplasm is the original site of DA production or if it leaks out of the vacuole is debatable (Kutchan et al. 1986).

## Physiological roles of DA in plant

**Fig. 2:**
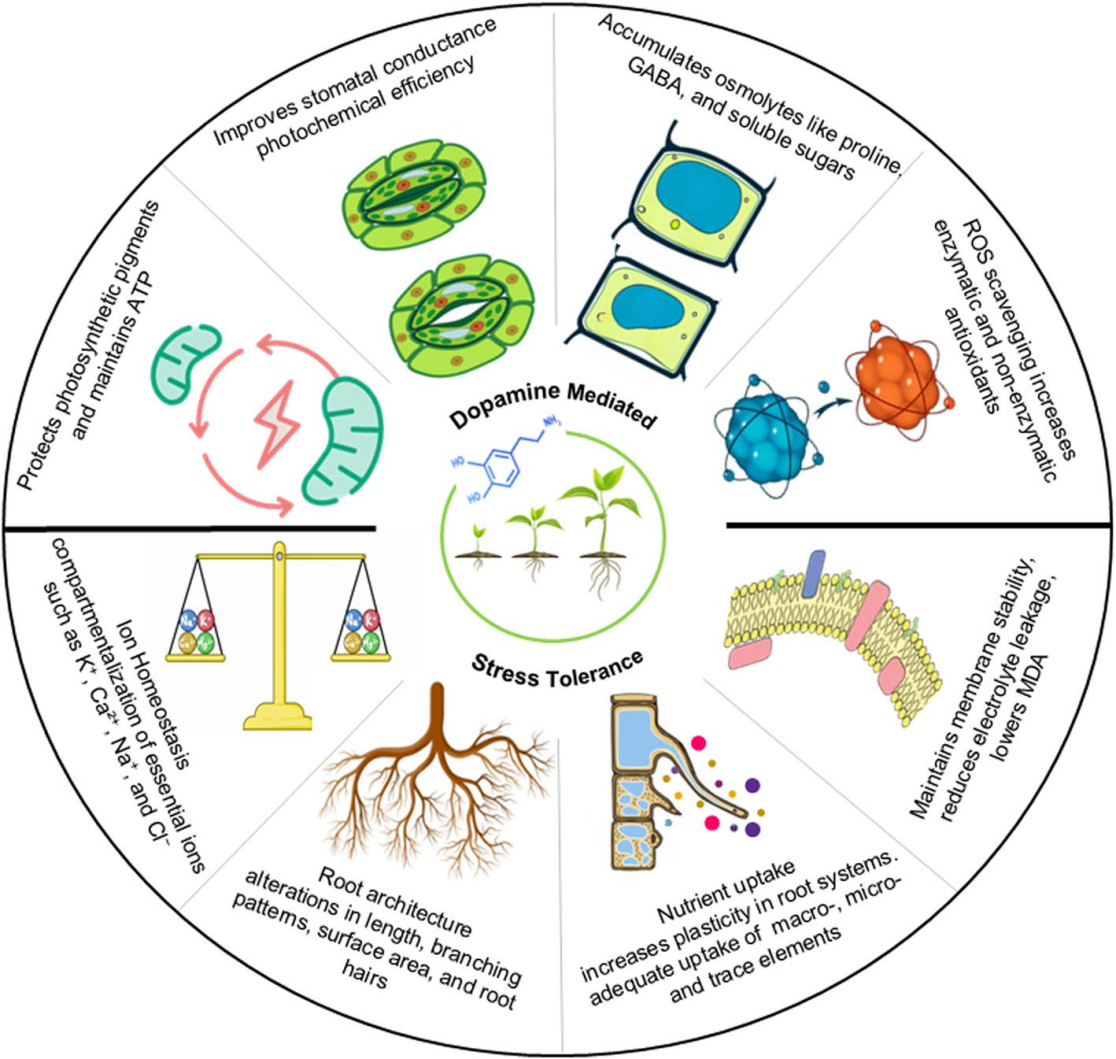
Effect of dopamine on plant physiology: Dopamine (DA) acts as a multifunctional regulator in plants by modulating essential physiological, biochemical, and cellular processes in stress tolerance. DA improves photosynthetic performance by enhancing stomatal conductance, chlorophyll stability, and photochemical efficiency, while also safeguarding mitochondrial integrity and ATP production. DA encourages the buildup of compatible osmolytes, such as proline, γ-aminobutyric acid (GABA), and soluble sugars, and boosts antioxidant defences to maintain reactive oxygen species (ROS) homeostasis. Furthermore, DA helps regulate membrane stability, ion balance, osmotic adjustment, and turgor maintenance, in addition to enhancing nutrient uptake and root system architecture—collectively supporting increased plant growth and stress resilience

In plants, DA regulates a broad spectrum of metabolic processes (Table 1). It contributes to stress adaptation by promoting, protecting, and altering photosynthesis, root system development, defense pathways, hormonal interactions, and water-nutrient acquisition, as described in Fig. 2. Through these coordinated adjustments, DA changes the way plants respond to stress conditions and maintain their normal growth and development.

**Table 1:** Effect of dopamine on different abiotic and biotic stress in plants at physiological, biochemical and molecular levels

S. No	Stress Type	Plant Species	Dopamine Treatment	Physiological Effects	Biochemical/Enzymatic Changes	Gene Expression Changes	References
1	Salt stress	*M. Domestica*	100 µM irrigation	↑ Photosynthesis, chlorophyll, stomatal aperture & density, uptake of P, S, Cu, and Mn; ↓ Uptake of Na⁺, Cl⁻	↑ APX, GR, GSH, MDHAR, DHAR, GSSG; ↓ ROS	↑MdHKT1, MdNHX1, MdSOS1	(Li et al. 2015)
2	Salt stress	Tomato	100 µM irrigation	↑ Plant-root dry weight, stem diameter, plant height, leaf area, total chlorophyll content, and Leaf relative water content ↓ Membrane permeability, ROS	↑ IAA, SOD, CAT, POD ↑ K^+^/Na^+^ and Ca^2+^/Na^+^ ↓ MDA, ABA	–-	(Yildirim et al. 2024)
3	Salt stress	Apple	100 µM irrigation in synergy with arbuscular mycorrhizal fungi	↑ Photosynthesis rate, root length, leaf surface area, average root diameter, root forks, stomatal conductance ↓ Membrane permeability	↑ Soluble carbohydrates, glucose, fructose ↑ K^+^, Na^+^, N, P ↓ Relative electrolyte leakage, MDA	↑ MdTYDC, MdSPS 1; 2; 6, MdCWINV1; 2, MdNINV 1;2, MdFLS, MdCHS, MdCHI	(Gao et al. 2020b)
4	Salt stress + Drought stress	Tomato	100 µM DA and 1 µM progesterone irrigation	↑ Relative water content, dry weight, tissue length ↓ Ion leakage, MDA	↑ Proline	↑ FeSOD, CAT2, GR1, P5CS, ACS2, APX1	(Akcay et al. 2024)
5	Salt stress	Soybean	100 µM or 200 µM	↑ Chlorophyll a, b, carotenoid, leaf area, shoot length, root length, marinated ions absorption,	↑ CAT, SOD, APX, POD, GR, ↑ P, Ca, N ↓ MDA, Na	↑ CAT, SOD, APX, POD, GR,	(Abo-Shanab and Diab 2024)
6	Drought	Soybean	Dopamine 100 µM and 24-epibrassinolide 100 nM	↑ Root length (RL), hypocotyl length (HL), and total length (TL) root epidermis thickness (RET), root endodermis thickness (RDT), root cortex thickness (RCT), vascular cylinder diameter (VCD), root metaxylem vessel (RMD) first count to germination (FCG), total germination (TG), and ↓ Mean time to germination (MTG)	↑ SOD, CAT, APX, and POX ↓ H_2_O_2_ and MDA	–-	(Pontes et al. 2024)
7	Drought	Apple	100 μM dopamine irrigated	↑ Seedling biomass, photosynthesis rates, chlorophyll concentrations, and stomatal apertures Trunk diameter (TD)	↑ Leaf relative water content (RWC) and hydrogen peroxide (H2O2) ↑ N, P, K, S, Cu, Zn, B, Ni, Mo, and Pb ↓ Ca, Mg, Fe, Mn, Al, Cr, As, and Cd	↑ SAG12 and PAO	(Liang et al. 2018)
8	Drought	Apple	100 μM Dopamne irrigated	↑ Photosynthetic pigments, net photosynthetic rate activating Ca^2+^ signaling pathways, stomatal conductance, water-use efficiency (WUEi)	↑ SOD, CAT, POD, APX, Chl a/b ratio ↓ ROS	CNGC and CAM/CML family genes TFs- WRKY, ERF, and NAC	(Gao et al. 2020b)
9	Drought	Apple	Dopamine 100 μM, irrigated	↑ Promote the absorption, utilization of nitrogen, plant length, stem diameter	↑ SOD, CAT, POD, APV, Endogenous dopamine, The nitrate reductase (NR), nitrite reductase (NiR), glutamine synthetase (GS), and ferredoxin-dependent glutamate synthase (Fd-GOGAT) ↓ MDA	NRTs: NRT1.1, NRT2.4, NRT2.5 and NRT2.7; (B) AMTs: AMT1.2, AMT1.5, AMT1.6 and AMT2.1	(Du et al. 2022a)
10	Low nitrogen stress	*M. hupehensis*	Dopamine 100 μM, irrigated	↑ Total root length, average diameter, root tip number, root volume, lateral root number, root surface area The root mass fraction (RMF), stem mass fraction (SMF) and leaf mass fraction (LMF)	↑ Activity of nitrate reductase, nitrite reductase, glutamic acid synthase and glutamine synthetase	Increases expression of ethylene signaling genes (ERF1, ERF2, EIL1, ERS2, ETR1, and EIN4)	(Liu et al. 2020b )
11	Nitrate Stress	cucumber	150 μM, hydroponic	↑ Fruit length, cross-sectional diameter, yield of the cucumber, root growth	↑ ACC, SOD, CAT, POD, APX, phosphorus, potassium in roots ↓ MDA	-	(Lan et al. 2022)
12	Nitrate Stress	cucumber	150 μM	↑ Shoot height, Stem diameter, Seedling index	↑ Chlorophyll content nitrate reductase (NR) glutamine synthetase (GS) and glutamate dehydrogenase (GDH) activity	CsSPS4, CsSUS3, CsNRT1.1	(Lan et al. 2020)
13	low nitrogen stress	Crisphead Lettuce	100 μM Spraying	↑ Vegetative growth, leaf area, number of outer leaves, fresh weight	↑ Chlorophyll content, % nitrogen and nitrates, vit C, total sugars ↓ MDA H_2_O_2_		(Farouk et al. 2023)
14	Cadmium stress	Apple	100 μM and 300 μM seed soaking	↑ Healthy vegetative growth	↑ Amino acid and phenols, chlorophyll content, root vigor ↓ Absorption of Cd in root and leaves, reduced ROS content	↑ Expression of detoxifying genes like HA7, NRAMP1, NRAMP3, HMA4, PCR2, ABCC1, MHX, NAS1, and MT2 ↓ expression of Cd transporter genes	(Zhang et al. 2023b)
15	Cadmium stress	Arabidopsis thaliana	100 μM, hydroponics	↑ Carotenoid, chlorophyll, and photosynthesis ↓Cd-induced chlorosis and necrosis,	↑ Ion homeostasis, ↓ Cd transporter genes	Regulated the expression of bHLH39, FRO2, IRT1, and NAS4	(Chang et al. 2025)
16	*Valsa mali*	Apple	100 μM, irrigated	↓ Tissue damage, length of lesions	↑ SOD, CAT. POD, PPO, Phenolic content, chitinase, and b-1, 3-glucanase, salicylic acid ↓ H_2_O_2_	↑ MdTYDC	(Liu et al. 2022a)
17	*Fusarium solani*	Apple	100 μM, irrigated	↑ Biomass. Root activity, photochemical efficiency ↓ Damage to root tissue	↑ DA, SOD, CAT, POD, PPO ↓ ROS	↑ Expression of chitinase, PR proteins, and β−1, 3-glucanase	(Liu et al. 2022b)
18	Chilling injury	Banana	150 μM, dipping	↑ Shelf life ↓ Reduced chilling injury symptoms	↑ Phenol and flavonoid biosynthesis, radical scavenging activity, endogenous proline, GABA ↓ ROS, MDA, PPO ProDH	↑ P5CS, OAT, SOD, CAT, GAD, GABA-T ↓ ProDH	(Nazari et al. 2024)
19	Chilling injury	Banana	150 μM, dipping	↑ Shelf life, chloro[hyll accumulation ↓ Reduced chilling injury symptoms	↑ Glycine betaine, SOD, CAT, APX, GR ↓ MDA, ROS, Electrolyte leakage	↑ TyrDC, CMO, BADH ↓ MDC, PPH, PaO, and RCCR	(Ali et al. 2023)
20	Chilling injury	Kiwifruit	150 μM, dipping	↑ Shelf life ↓ Reduced chilling injury symptoms	↑ SUMO E3 ligase, ATP. NADPH, Salicylic acid ↓ Poly (ADP-Ribose) polymerase 1, and sucrose nonfermenting 1-related kinase 1, MDA, ROS, and Electrolyte leakage	↑ Target of rapamycin TOR, SIZ1, SOD, CAT, PLD, LOX ↓ PARP1, SnRK1	(Aghdam et al. 2025)

*Abbreviations*: *ABA* abscisic acid, *ACC* 1-aminocyclopropane-1-carboxylic acid, *AMT* ammonium transporter, *APX* ascorbate peroxidase, *BADH* betaine aldehyde dehydrogenase, *CAM/CML* calmodulin/calmodulin-like proteins, *CAT* catalase, *CMO* choline monooxygenase, *CNGC* cyclic nucleotide-gated channel, *DHAR* dehydroascorbate reductase, *ERF* ethylene response factor, *Fd-GOGAT* ferredoxin-dependent glutamate synthase, *GABA* γ-aminobutyric acid, *GABA-T* γ-aminobutyric acid transaminase, *GAD* glutamate decarboxylase, *GR* glutathione reductase, *GSH* reduced glutathione, *GSSG* oxidized glutathione, *H₂O₂* hydrogen peroxide, *IAA* indole-3-acetic acid; LOX, lipoxygenase, *MDA* malondialdehyde, *MDHAR* monodehydroascorbate reductase, *NAC* NAC transcription factor, *NiR* nitrite reductase, *NR* nitrate reductase, *NRT* nitrate transporter, *OAT* ornithine aminotransferase, *PaO* pheophytin pheophorbide *a* oxygenase, *PARP1* poly(ADP-ribose) polymerase 1, *P5CS* Δ^1^-pyrroline-5-carboxylate synthetase, *PLD* phospholipase D, *POD* peroxidase, *PPO* polyphenol oxidase, *PR* pathogenesis-related, *ProDH* proline dehydrogenase, *RCCR* red chlorophyll catabolite reductase, *ROS* reactive oxygen species, *SIZ1* SUMO E3 ligase 1, *SnRK1* sucrose non-fermenting 1-related protein kinase 1, *SOD* superoxide dismutase, *TOR* target of rapamycin, *WRKY* WRKY transcription factor; ↑ increase; ↓ decrease

### Enhancement of photosynthetic efficiency

Reduced photosynthesis is a hallmark of unfavourable conditions (Sharma et al. 2020). Plants under stress usually produce ROS as a signal for response; however, an excess of ROS causes cellular damage, oxidizing pigments crucial for light absorption (Gao et al. 2020b; Liang et al. 2018). This disruption in the photosynthetic apparatus hampers the plant's ability to assimilate carbon, leaving it with limited energy (Akcay et al. 2024; Ji et al. 2022). Plants thus attempt to conserve their energy by adjusting their stomatal behavior and conductance, which ultimately results in stunted growth and reduced yield. (Ji et al. 2022; Nguyen et al. 2023).

Dopamine is found to alleviate the photosynthetic rate in plants under stress (Gao et al. 2020a, 2020b, 2021a; Liang et al. 2018; Liu et al. 2025). It ensures the stability of the light-dependent reaction and supports optimum light absorption. (Lan et al. 2020). It was also found to maintain stomatal conductance, resulting in well-regulated photosynthesis, optimal transpiration, and higher bioaccumulation compared to the untreated plants. DA reinforces energy transduction and helps plants maintain their performance and growth under challenging conditions (Table 1) (Liu et al. 2020a).

### Regulation of root architecture

Root system is a primary interface between the plant and the soil environment (Li et al. 2015). Therefore, its structural vitality is crucial for acquiring the nutrients needed for growth. Plants under stress often demonstrate a detrimental change in root architecture, failed gravitropic response, and poor root-microbiome interaction (Cao et al. 2024). Stress-induced alterations in the root system causes poor nutrient acquisition, reduced water uptake, disrupted hormone balance, and impaired photosynthesis (Ding et al. 2024; Guidotti et al. 2013).

The application of exogenous DA helps protect roots from the ill effects of stress, providing optimum nutrient absorption for plants (Table 1). (Gao et al. 2020a; Li et al. 2015; Liang et al. 2017),

It enables the transport of essential nutrients needed for normal plant growth and maintenance, and stimulates root vigor to explore water (Li et al. 2015). Such transformations indicate that DA application in plants reinforces the below-ground systems of plants to cope with stress as an adaptive strategy (Liang et al. 2017; Liu et al. 2025; Chapman et al. 2012). Dopamine is also produced and secreted by the roots as a major exo-metabolite that functions as a bioactive component of root exudates, suggesting a role in the root-soil-microbe interface (Ding et al. 2024). DA also interacts with hormones responsible for root growth; however, the role of DA changes depending on the type of stress and the species of plant, indicating its species-specific and stress-specific function (Guidotti et al. 2013).

### Activation of antioxidant defence mechanisms and Promotion of secondary metabolites

As discussed earlier, the -OH group located on the benzene ring contributes to DA’s ability to scavenge free radicals (Wakamatsu et al. 2019). This antioxidant activity enables DA to protect membrane-bound cell organelles, such as mitochondria, to prevent excess electron leakage, the nucleus to protect genetic integrity, the plasma membrane, which acts as a first line of defence, vacuoles to ensure turgor pressure, and the endoplasmic reticulum for protein synthesis (Yen and Hsieh 1997; Hou et al. 2016). Thus, by protecting these membranes and membrane-bound cell organelles, DA maintains the optimal functioning of cellular activities (Huang et al. 2024; Yen and Hsieh 1997). Along with the catechol moiety, DA also enhances the redox potential of plant cells by improving the activity of antioxidant enzymes responsible for scavenging free radicals and converting them into less harmful species or water molecules (Ahammed et al. 2020, 2025). Usage of DA is thus found to ensure normal, stable, functional, and more resilient cells under stress (Abdulmajeed et al. 2022; Zhang et al. 2023a). In addition to the increased antioxidant defence, DA stimulates the biosynthesis of secondary metabolites like phenol and flavonoid (Bala 2020). The synthesized metabolites then act as a source of lignin, giving strength to the cell wall (Soares et al. 2007). The phenols and flavonoids take part in scavenging activities as a non-enzymatic antioxidant power (Aghdam et al. 2025). Apart from this, DA also initiates the accumulation of proline and glycine betaine (Table 1), which have a role in osmoregulation in plants, and polyamines, which stabilize the cell membrane (Jiao et al. 2021; Ali et al. 2023).

### Interaction with plant hormonal networks

Plants heavily rely on hormonal crosstalk for adaptation to their constantly changing environment; however, the prolonged stress causes this system to collapse, leading to damage. As a neurotransmitter-like molecule in plants, DA functions as an important modulator within the plant hormonal network, influencing the biosynthesis, degradation, and signalling of multiple phytohormones during stress (Elstner et al. 1976). It interacts with hormones like auxin (Guidotti et al. 2013), gibberellic acid (Zhang et al. 2023b), melatonin (Chakraborty et al. 2022), abscisic acid (Yildirim et al. 2024), jasmonic acid (Table 1) (Zhang et al. 2023b), ethylene (Liu et al. 2020b), and it also impacts its own biosynthesis upon application. Dopamine is also capable of affecting the transcription of genes involved in the hormone biosynthesis, like indole-3-acetic acid (IAA), contributing to a fine-tuned turnover of stress-related phytohormones (Yildirim et al. 2024). It also helps in hormonal regulation through coordinated hormone signalling, maintaining metabolic stability under a challenging environment.

### Improvement of water and nutrient absorption efficiency

The application of DA enhances the nutrient resorption efficiency and water-holding capacity of the plant (Yanosky 2005; Liu et al. 2020a). When applied exogenously, DA increases the concentration, transport, and uptake of nutrients, leading to an altered distribution within the plant cell (Liang et al. 2018). The increased absorption of water molecules by the cell will provide a healthy turgor pressure, which in turn drives nutrient transport. It facilitates the closing and opening of stomata, ensuring proper transpiration. This water and nutrient absorption efficiency, in turn, maintains root cell rigidity, as well as apoplastic and symplastic pathways, ensuring the efficient flow of minerals within and across the membrane (Liang et al. 2017). This movement causes a gradient difference, facilitating the cytoplasmic streaming or cyclosis, giving improves stress tolerance (Ahammed and Li 2023).

## DA and plant stress responses

### Abiotic stress

**Fig. 3:**
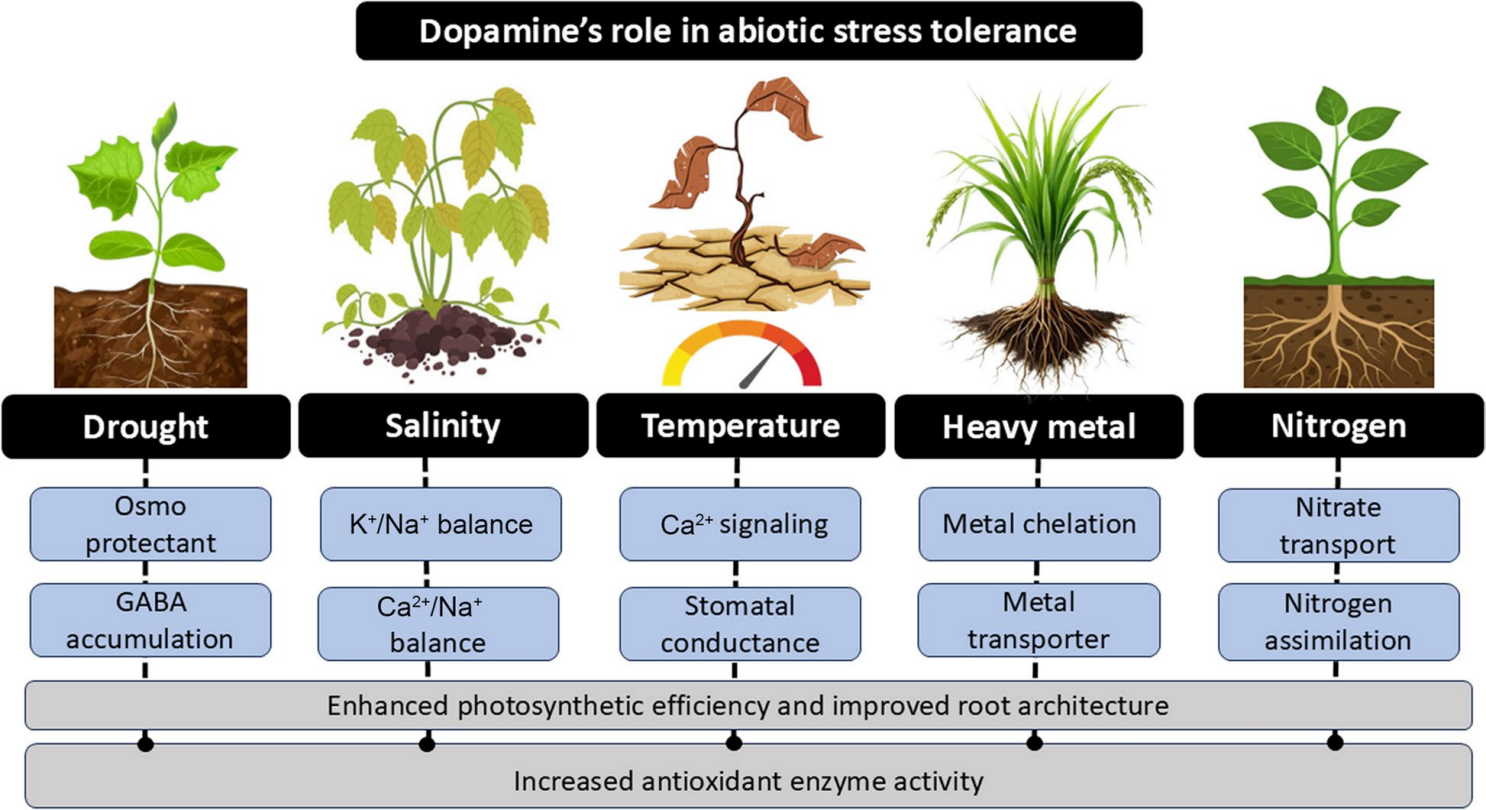
Role of dopamine (DA) in abiotic stress tolerance in plants. DA mediates plant adaptation to various abiotic stresses by activating specific physiological and metabolic responses. During drought stress, DA promotes osmotic adjustment through the accumulation of osmoprotectants and γ-aminobutyric acid (GABA). Under salinity stress, DA helps maintain ionic balance by regulating potassium (K⁺) and sodium (Na⁺) levels. In response to temperature stress, DA primarily enhances stomatal conductance and calcium (Ca^2^⁺) signalling. When faced with heavy metal stress, DA supports metal chelation and detoxification via transporters, while under nitrogen stress, it improves nitrate transport and nitrogen assimilation. Overall, these responses boost antioxidant enzyme activity, photosynthetic efficiency, and root plasticity, improving the plant’s overall stress tolerance

Abiotic stress, such as drought, salinity, extreme temperatures, nitrate stress, and heavy metals, disrupts the cellular homeostasis, limiting the plant growth and productivity. In plants, as shown in Fig. 3, DA accumulates under stress and performs a multifunctional role like modulation of redox homeostasis, recruiting beneficial microbes for nitrogen fixation, maintaining membrane integrity, and supporting efficient photosynthetic and metabolic processes.

#### Drought

Prolonged drought has caused plants to make adaptive modifications due to water scarcity (Bandurska 2022). They close their stomata during drought to preserve water, but, this also hinders the photosynthesis efficiency of the plant (Detto and Pacala 2022). Thus the sum total of energy available for normal cellular functioning of the cell gets affected due this altered stomatal behaviour (Garcia et al. 2023). Malfunctioning of this cellular machinery leads to plant stress which is characterized by an overproduction and accumulation of ROS such as H_2_O_2_, OH^−^, and O_2_^−^. Drought also restricts the absorption and movement of nitrogen withing the cell due to compromised transpiration, impaired xylem transport and reduced root metabolic activity. Root can take up some amount of nitrogen but it usually accumulates due to reduction in nitrate and ammonium transporting enzymes. The buildup in ROS further damages the root membrane integrity limiting the survival of plant (Du et al. 2022a).

DA primarily protects the photosynthesis efficiency of plants as a central plot for mitigating drought stress. (Liang et al. 2018) highlighted the use of DA to reduce the drought-related degeneration of photosynthetic pigments and enhance the CO_2_ assimilation, which leads to an enhanced photochemical efficiency in apple (Ahammed and Li 2023). A 100 μM DA treatment in the apple plant has enhanced net photosynthetic rate, relative water content, stomatal conductance, and photosynthetic pigments such as chlorophyll a and b (Gao et al. 2021b, 2020b; Wang et al. 2021).

Known for its remarkable antioxidant capacity, DA effectively mitigates oxidative stress (Etemadi et al. 2018). It does so by lowering the ROS accumulation (Gao et al. 2020a), primarily through the activation of antioxidant enzymes like CAT, SOD, APX, GR (Akcay et al. 2024). Treatment with DA has resulted in upregulated transcripts of *SOD, CAT, APX, GR* has been seen in plants like apple (Gao et al. 2020b), cucumber (Lan et al. 2022), crabapple (Liang et al. 2017), tomato (Ahammed et al. 2025; Yildirim et al. 2024). The transcriptomic analysis of the apple plant after application of 100 μM DA revealed an upregulation of calmodulin and calmodulin-like genes (*CaM/CML*) and 2 *CNGC* involved in Ca^2+^ signalling (Table 1). The transient elevation in cytosolic calcium during stress initiates a cascade of reactions leading to an adaptive cellular response (Tuteja and Mahajan 2007), which will further increase the downstream transcription factors like *MYBs* and *WRKYs* (Akula and Mukherjee 2020). The elevated expression of CaM protein will also enhance the antioxidant defence system in plants as SOD is CaM-dependent enzymes (Arora et al. 2024) and the activation of Glutathione peroxidase (*GPX*) is also linked with CaM (Ming and Zhong Li 1995).

Water-scarce condition often induces morphological changes in roots, and DA treatment contributes to enhanced root development and elongation for efficient uptake of water and essential nutrients (Liang et al. 2018). When applied along with 100 nM 24-epibrassinolide (EBR), a 100 µM DA impacted the anatomy of roots, changing its epidermis tissue, vascular cylindrical tissue, and metaxylem. Root architectural optimization could be one of the strategies that DA deploys for the drought stress. However, in this experiment, soybean seedlings treated only with DA yielded better results, indicating that EBR might hinder the mitigating efficiency of DA in a water-scarce environment (Pontes et al. 2024). In synergy, equal concentration of 100 μM melatonin and DA helps in influencing and fixing of root microflora by recruiting some beneficial rhizosphere microbial communities to favour N_2_ fixation in apple plant. The treatment increased the operational taxonomic unit (OTU) in plant root favouring the fungal diversity than bacterial as fungal OTU as they tolerates low moisture better and also their hypae can access water in micropores. Besides this DA and Melatonin also upregulated the gene expression of nitrate and ammonium transport gene facilitating the nitrogen transport (Table 1). It upregulates genes such as *NRT1.1, NRT2.4, NRT2.5, NRT2.7 AMT1.2, AMT1.5, AMT1.6, AMT2.1,* (Du et al. 2022a). Dopamine functions in plants as both a direct and indirect antioxidant agent, contributing to the scavenging of reactive oxygen species and the activation of endogenous antioxidant defence systems.

#### Salinity

High salt content of the soil hinders plant growth and development. Excess salinity leads to reduced water absorption by roots due to a lower soil water potential, resulting in leaf wilting, reduced cell expansion, and slower growth. Ion toxicity due to high Na^+^ and Cl^−^ ions damages the cell membrane and causes a nutrient imbalance. High accumulation of Na^+^ competes with other essential nutrients, such as potassium, calcium, and magnesium, causing poor enzyme activity, a weaker cell wall, and disrupted metabolism. The excess of ROS leads to DNA damage and protein denaturation (Bala 2020). Salinity is also found to cause reduced root length and branching, as well as lower root hair development, due to impaired nutrient uptake (Abdelkader et al. 2012).

Studies in recent years highlighted DA’s exogenous application and its ability to mitigate salt stress in plants like tomato (Yildirim et al. 2024), apple (Gao et al. 2020a), rice (Abdelkader et al. 2012), and *M. hupehensis* (Li et al. 2015). An irrigation treatment of 100 µM DA in apple and tomato seedlings significantly improved growth, dry root weight, stem diameter, plant height, and the area of the leaf (Li et al. 2015; Yildirim et al. 2024). Ion homeostasis is crucial for plant survival under saline conditions. DA improves the uptake and transport of ions, favouring the accumulation of nutrients and osmotic balance (Bala 2020; Liu et al. 2020a). Exogenous DA 100 μM in *M. hupehensis* inhibits excess Na and Cl uptake and enhances the acquisition of minerals like K, N, P, S, Cu, and Mn, indicating improved ion balance in both pot experiment and hydroponically grown seedlings. The upregulation of genes such as *MdHKT1, MdNHX1*, and *MdSOS1* elucidates the function of DA in preventing excess Na from reaching the leaves, supporting turgor pressure by pumping Na^+^ into the vacuole, and expelling Na^+^ out of the cell via the root by activating the salt overly sensitive pathway, which is a core salt defence system in plants (Li et al. 2015).

A dose of 100 μM DA also alleviates the negative effects of salt stress in tomato seedlings by maintaining the Ca_2_^+^/Na^+^ and K^+^/Na^+^ ratios in the plant, increasing the root dry weights by 286.84%, plant height by 108.37%, and leaf area by 158.28%, respectively, compared to the control. Additionally, DA reduces membrane leakage of electrolytes by maintaining the integrity of the lipid bilayer through increased activity of antioxidant enzymes such as CAT, SOD, and POD. It also reduces the rise in ABA in tomato plants (Table 1), facilitating effective transpiration and photosynthetic capacity, and also enhances the IAA content (Yildirim et al. 2024). Similarly, in the apple plant, 100 μM DA acts in synergy with *arbuscular mycorrhizal fungi* (AMF), increasing the absorption of N, P, and K in roots and reducing the relative electrolyte leakage as compared to the control plant. The combination maintained plant cell membrane stability and improved photosynthesis by increasing root length, surface area, average diameter, and number of root forks, which increased the surface area to absorb nutrients and water under salt stress (Gao et al. 2020a). The metabolomic analysis of a halophytic grass *Puccinella nuttalliance* revealed an increased level of endogenous DA and proline when treated with exogenous DA (Vaziriyeganeh et al. 2021). Also in *Glycine max* (L.) Merr. Plant DA marinated the ion absorption and triggers the accumulation of glycine betaine and proline, along with soluble sugars and proteins (Abo-Shanab and Diab 2024).

#### Heavy metals

The contamination of agricultural soil by heavy metals primarily originates from anthropogenic activities, threatening food safety and the environment simultaneously (Sharafi and Salehi 2025). Among these heavy metals, chromium Cr, Cd, and Pb show high toxicity in soil, disturbing the normal functioning of the plants (Khatun et al. 2022; Srivastava et al. 2021). Studies across plant species, such as tomato (Ahammed et al. 2025), apple (Cao et al. 2023b), common bean (Abdulmajeed et al. 2022), and duckweed (Wang et al. 2023) demonstrated DA’s application in effectively mitigating the detrimental effects of heavy metal stress (Table 1).

In the tomato plant, a dose of 100 μM DA mitigates Cr stress by activating NADPH oxidase, which is responsible for H_2_O_2_-based signaling. Here, the exogenous application of H_2_O_2_ was also found to mimic the protective effect of DA, while the application of DPI abolished the DA-induced stress tolerance, demonstrating that H_2_O_2_ signalling is a key mediator of DA-induced resilience in tomato plants. DA upregulates the antioxidant genes like *SOD (Cu–Zn SOD), CAT1, APX, POD,* and *GR1,* metal chelating genes such as *PSC and GSH,* strengthening the oxidative boost, metal chelation, and reducing the Cr accumulation (Ahammed et al. 2025).

The richness of the rhizosphere community, such as *Pseudoxanthomonas*, *Aeromicrobium, Frankia, Novosphingobium*, *Bradyrhizobium*, *Saccharimonadales, and Streptomyces,* has been found to be enhanced in apple seedlings with a treatment of 100 μM DA under Cd stress. The network analysis of the microbial community showed a change in its composition, favoring a shift in the keystone species to resist Cd in plants, suggesting that DA alleviated Cd stress by recruiting beneficial microbes and enhancing the physiological resilience of the plant (Cao et al. 2023b). In addition to its role in heavy metal stress, DA also interacts synergistically with other plant growth regulators and antioxidants (Akcay et al. 2024). For instance, in common bean plants exposed to Cd stress, the combined application of DA and silymarin (Sm) exhibited a synergistic effect by reducing oxidative stress and enhancing both enzymatic and non-enzymatic antioxidant activities (Abdulmajeed et al. 2022). The application of 200 µM exogenous DA regulates the intracellular Ca^2+^ signalling in duckweed, preserves the photosynthetic apparatus, reactivates the shikimic acid pathway, and electron transport for optimum energy. (Wang et al. 2023). DA also mediates ion homeostasis and detoxification. Transcriptomic and metabolomic analyses under cadmium (Cd) stress have revealed that DA treatment upregulates genes such as *FROs* (ferric reductase oxidases), *HIPPs* (heavy metal-associated isoprenylated plant proteins), and *LHC* (light-harvesting complex proteins). These genes contribute to Cd uptake regulation and detoxification processes, underscoring DA’s role in modulating metal transporter systems and mitigating oxidative damage (Zhang et al. 2025).

In the case of pollutants like bisphenol A in cucumbers, DA enhances the overall energy charge of the cell, root vitality, and nutrient absorption. The DA also enhanced scavenging activity by stimulating the glutathione-ascorbate cycle, resulting in lower MDA and ROS accumulation. It also promoted BPA metabolism, reducing its accumulation in leaves and roots (Ahammed et al. 2020). L-DOPA has shown exemplary tolerance toward the Cd stress in Arabidopsis, suggesting that even the intermediate in DA synthesis can also modulate distinct pathways. These findings underscore the interactions between signalling molecules and their roles in plant stress responses, underscoring the need for further research to fully elucidate the underlying mechanisms (Chang et al. 2025). These findings elucidate DA as a molecular regulator of transporter gene networks. By reprogramming nutrient and ion transporter activity, DA not only supports elemental homeostasis but also reinforces metabolic flexibility and stress resilience in plants.

#### Nitrogen stress

Regardless of its form, such as nitrate (NO_3_^−^) and ammonium (NH_4_^+^), nitrogen acts as a stress for plants (Lan et al. 2022). However, the DA application has been found to ease this stress effectively. For instance, in an excess nitrate-stressed cucumber, the root irrigation treatment with 150 µM DA enhances the root growth, photosynthesis, and antioxidant activity, and restricts the cellular level damage, keeping the overall fruit quality intact (Lan et al. 2022). DA also plays a scavenging role in curtailing the oxidative stress generated by the N_2_ imbalance in plant species. For instance, in cucumber, excess nitrate leads to an overproduction of MDA and superoxide anion (Lan et al. 2022) is curtailed by promoting enzymes such as SOD, CAT, and APX (Ahammed and Li 2023).

Another molecular-level study for nitrate stress in cucumber suggests that 150 µM DA enhances the activities of enzymes such as sucrose phosphate synthase, acid invertase, and nitrate reductase by upregulating the gene *CsSPS4, CsNR1, and CsSUS3,* resisting nitrate toxicity by coordinating photosynthesis and carbon–nitrogen metabolism (Lan et al. 2020). A dose of 100 µM DA in hydroponically grown *M. hupehensis* improves the uptake of phosphorus, nitrogen, and potassium (Liu et al. 2020b) and boosts enzymes linked to nitrogen assimilation and regulates ethylene biosynthesis, suggesting a DA-ethylene-mediated nitrogen stress response (Du et al. 2022b). As the ability of DA in root modification has already been discussed, it impacts the fixation of N_2_ in low nitrate or ammonium stress. However, the DA application mitigated these changes, promoting root growth and enhancing the uptake of NO_3_^−^ and NH_4_^+^ (Du et al. 2022b). This suggests that DA plays a significant role in optimizing root morphology for improved N_2_ acquisition under stress. In *M. hupehensis*, DA application increased the expression of nitrate transporter genes *NRT1.1*, *NRT2.4*, *NRT2.5*, and *NRT2.7* under nitrate-deficient conditions (Duet al. 2022a). Similarly, in apple, DA biosynthesis, enhanced N_2_ deficiency tolerance by upregulating carbohydrate-related genes and *MdORG2*, a transcription factor that activates *MdTyDC* expression (Table 1) (Liu et al. 2025).

#### Temperature stress

Scavenging the ROS and maintaining membrane integrity are important functions in mitigating cold temperature stress in plants. Plants like watermelon (Jiao et al. 2021) and grape (Zhou 2025), have been found to successfully sustain this stress with the use of DA 100 µM and 400 µM, respectively. In watermelon seedlings, treatment of 100 µM DA results in accumulation of endogenous polyamine, osmolyte modulations, and improved antioxidant system (Jiao et al. 2021). In the grape plant, DA also plays the role of an osmolyte regulator and ion balancer, thereby maintaining cell vitality. It also enhances the overall antioxidant system. It also enhanced the overall antioxidant system and increased the photosynthetic pigments (Zhou 2025).

Whereas in mitigation of postharvest CI, DA promoted the phenylpropanoid pathway, produced more phenols and flavonoids (Nazari et al. 2024). This will lead to higher antioxidant scavenging activities shown as higher DPPH, FRAP, ABTS content, supplemented with lower activity of polyphenol oxidase (*PPO*) (Aghdam et al. 2025; Ali et al. 2023; Nazari et al. 2024). In Addition, DA treatment of 150 µM in banana fruits raises the endogenous proline concentration by stimulating the activity of proline-synthesizing enzymes such as pyrroline-5-carboxylate synthetase (*P5CS*) and ornithine δ-aminotransferase (*OAT*), indicating its role in osmotic regulation (Nazari et al. 2024). Along with this application of DA also found to enhance endogenous γ-aminobutyric acid (GABA) and glycine betaine accumulation which supports the cell by participating in osmoregulation, ROS detoxification, ion homeostasis and macromolecule protection in banana and kiwi fruits subjected to chilling stress (Aghdam et al. 2025; Ali et al. 2023). DA significant mitigated the CI in banana stored at 7 ºC for 21 days and kiwifruits stored at 1 °C for 120 days by activating phenylpropanoid pathway, endogenous proline, GABA, glycine betaine content, enhancing antioxidant defence system, lower electrolyte leakage, H_2_O_2_ and MDA, and maintaining membrane integrity (Aghdam et al. 2025; Ali et al. 2023; Nazari et al. 2024).

### Biotic stress

**Fig. 4:**
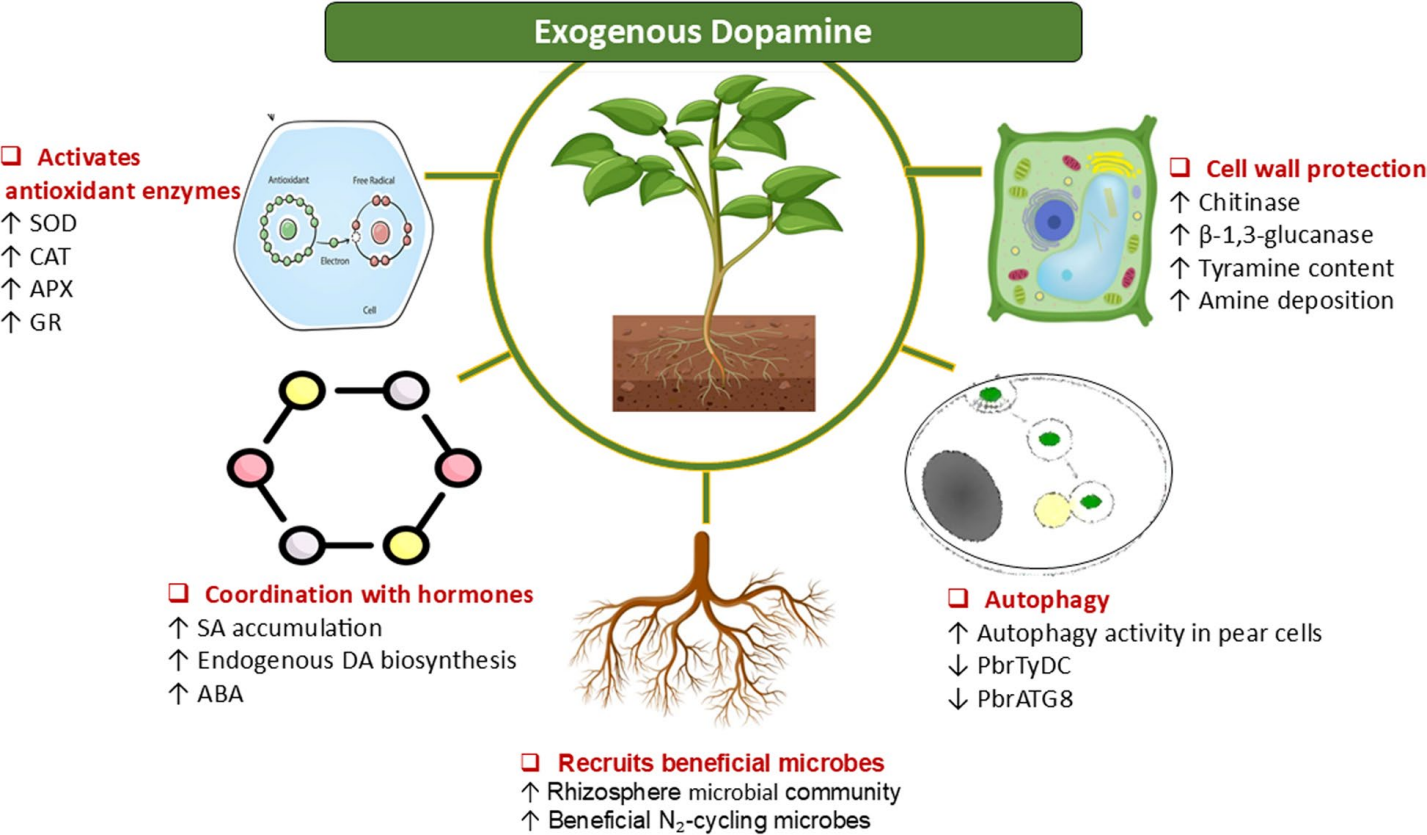
Role of DA in biotic stress: Exogenous dopamine (DA) boosts plant immunity by activating antioxidant defence enzymes, including superoxide dismutase (SOD), catalase (CAT), ascorbate peroxidase (APX), and glutathione reductase (GR), thereby reducing pathogen-induced oxidative stress. DA also promotes endogenous DA production by inducing tyrosine decarboxylase (*TYDC*) and helps coordinate hormone-dependent defence signalling by modulating salicylic acid (SA) and abscisic acid (ABA) pathways. Cellular protection is further strengthened through the deposition of callose, lignification, the accumulation of phenolics, and the accumulation of amines in the cell wall. Additionally, DA triggers autophagy by regulating autophagy-related ( ATG) genes and attracts beneficial rhizospheric microorganisms, collectively enhancing structural barriers, metabolic defences, and resistance to pathogens infection

In plant systems, DA accumulation is associated with enhanced resistance to pathogen infection through the reinforcement of antioxidant capacity, activation of defence-related genes, and strengthening of cell wall barriers, as shown in Fig. 4. Exogenous DA application has been reported to reduce disease severity by limiting oxidative stress, promoting hydrolytic enzymes involved in pathogen degradation, and coordinating hormone-dependent defence signalling.

A prominent example of DA's role in mitigating biotic stress is observed in apple replant disease caused by Fusarium solani. The exogenous application of 150 μM DA was found to enhance the biosynthesis of endogenous DA and effectively reduce root system damage caused by *F. solani*. This effect was observed with enhanced antioxidant enzymes, which thereby reduce oxidative stress and ROS accumulation. The application of DA was also found to have upregulated certain defence-related genes that code for chitinase and β−1,3-glucanase. The plant also has shown a bolstering resistance by an increased tyramine content and cell wall-bound amine deposition (Liu et al. 2022b).

Similar results were found in the apple plant with infection by *V. mali,* where the accumulation of chitinase and β−1,3-glucanase activity was observed again (Table 1). Resistance to infection here was acquired through an upregulated expression and accumulation of phenolic content and salicylic acid (SA) (Liu et al. 2022a). In pear ring rot infection, a foliar spray with 100 µM DA imparted resistance against *Botryosphaeria dothidea* by enhancing the autophagy activity in pear cells. It silences the genes like *PbrTyDC* and *PbrATG8* responsible for reduced resistance to the infection in pear. This observation highlights the importance of DA in boosting plant immunity as well (Zhang et al. 2023c). Thus, DA also plays a role in modulating the gene expression of different defence related genes acting as a multi-regulatory molecule in plants.

In potato, catecholamine levels, including DA has increased significantly via (*TyDC*). Furthermore, catecholamines are involved in starch-sucrose conversion and are induced under various stress conditions, including ABA treatment. These findings underscore the potential of catecholamines as novel stress agents in plants (Świędrych et al. 2004).

Phloridzin, an allelochemical that inhibits apple growth and disrupts rhizosphere microbial communities, can be effectively counteracted by DA application. DA reduces phloridzin-induced growth inhibition by lowering ROS levels and enhancing N_2_ transport. Moreover, DA positively influences the rhizosphere microbial community, enriching beneficial N_2_-cycling microbes. This is achieved by enhancing soil N_2_ degradation and fixation (Du et al. 2024).

## Current knowledge gaps and challenges

Despite positive research findings, significant knowledge gaps and challenges persist regarding the use of DA in plant stress. The narrow crop diversity explored so far focuses on crops such as *M. hupehensis* (Li et al. 2015; Liang et al. 2017; Liu et al. 2020b; Gao et al. 2021b; Du et al. 2022c, b; Zhang et al. 2023a), tomato (Yildirim et al. 2024; Akcay et al. 2024; Prestes et al. 2025; Ahammed et al. 2025), duckweed (Wang et al. 2023), and cucumber (Ahammed et al. 2020; Ji et al. 2022; Lan et al. 2022), while many economically important crops, particularly tropical fruits, legumes, and cereals, remain uninvestigated. The mitigation of CI in banana (Ali et al. 2023; Nazari et al. 2024) and kiwifruit (Aghdam et al. 2025) demonstrated the potential application of DA in postharvest treatment. Yet such reports are still rare, and mechanistic insights remain limited. Transcriptional and translation level studies in DA transporters, receptors, and Transcription factors are not well understood. Unlike animals, where DA receptors are well established, the identification and characterization of specific DA receptors in Plants has not been done yet (Lachowicz and Sibley 1997; Skirycz et al. 2005; Liu et al. 2020b). This gap hinders a full understanding of how DA is perceived and transduced into physiological responses. Furthermore, the dose-dependent nature of DA’s effects presents both an opportunity and a challenge. While 100 µM appears effective in many contexts (Gao et al. 2020b; Jiao et al. 2021; Li et al. 2015), the threshold for phytotoxicity is not well defined, and little is known about the consequences of long-term or high-dose exposure.

This absence of toxicological data brings up safety concerns, more importantly, when DA is utilized in commercial agriculture or during postharvest handling. Currently, there is no regulatory framework outlining maximum permissible limits, or legal guidelines for exogenous DA use in agriculture. Addressing these gaps by conducting multi-species validation, expanding postharvest research, exploring molecular-level investigations, profiling dose–response relationships, and performing safety assessments is important for practical agricultural and postharvest solutions.

### Future perspectives

Research on DA’s application in plant stress holds promise in the context of adverse climatic effects on the plant system and prolongation of shelf life and safe transport. Through the biotechnological advancement, such as genetic engineering, increasing the endogenous DA content by overexpressing the key gene (*TyDC*) can increase DA content in the plant, making it climate resilient. Such innovation replace the repeated exogenous DA applications. The postharvest application of DA offers a new way for further research. Novel opportunities to reduce CI in chilling-sensitive tropical fruits like banana, kiwifruit, and mango could contribute to reducing postharvest losses, especially under cold storage conditions.

Furthermore, DA may be utilised with other plant growth-promoting strategies, including phytohormones (e.g., melatonin, brassinosteroids), beneficial microbes, and nutrient management practices. These synergistic approaches could give a better stress mitigation through enhanced antioxidant defence, energy metabolism, and signaling crosstalk. As research goes on, integrating DA into holistic crop management strategies may become an important component of next-generation sustainable agriculture aimed at ensuring food security in a changing climate.

## Conclusion

DA has come up as a modulator in plant responses during stress. Its use has consistently shown positive results in responses like redox homeostasis, nutrient regulation, osmotic adjustment, salinity stress, and overall plant resilience. Especially, its effectiveness in mitigating postharvest CI and elongating the shelf life of fruit further underscores its utility beyond the field and also extends into storage and marketability. However, a deep understanding of DA’s signalling in stress response and its in-depth mechanistic studies are very important. Mechanism of DA with hormones like melatonin that are antagonistic in animals but act in synergy in plant stress could be explored as well. With focused research and innovation, DA might become a key molecule of sustainable crop management strategies, supporting agricultural productivity in the face of global climate challenges.

## Data Availability

There is no additional associated data with this article.

## Abbreviations

APX: Ascorbate peroxidase
ATP: Adenosine triphosphate
Cam: Calmodulin
CAT: Catalase
Cd: Cadmium
CI: Chilling injury
CML: Calmodulin-like
CO_2_: Carbon dioxide
Cr: Chromium
DA: Dopamine
DDC: DOPA decarboxylase
EBR: Epibrassinolide
ERF: Ethylene response factor
FROs: Ferric reductase oxidases
GABA: γ-Aminobutyric acid
GR: Glutathione reductase
H_2_O_2_: Hydrogen peroxide
HAT: Hydrogen atom transfer
HIPPs: Heavy metal-associated isoprenylated plant proteins
IAA: Indole acetic acid
L-DOPA: Levodopa
LHC: Light-harvesting complex proteins
MAPKs: Mitogen-activated protein kinases
MDA: Malondialdehyde
MH: Monophenol hydroxylase
N_2_: Nitrogen
NADPH: Nicotinamide adenine dinucleotide phosphate
-NH_2_: Primary amine group
NH_4_^+^: Ammonium
NO_3_^−^: Nitrate
O^2^^−^: Superoxide ion
-OH: Hydroxyl group
P5CS: Pyrroline-5-carboxylate synthetase
Pb: Lead
POD: Peroxidase
PPO: Polyphenol oxidase
RBOH1: Respiratory burst oxidase homolog
ROS: Reactive oxygen species
SA: Salicylic acid
SET: Single electron transfer
SOD: Superoxide dismutase
TF: Transcription factor
TH: Tyrosine hydroxylase
TyDC: Tyrosine decarboxylase
